# Serum Levels of Proinflammatory Cytokines in Painful Knee Osteoarthritis and Sensitization

**DOI:** 10.1155/2015/329792

**Published:** 2015-03-02

**Authors:** Marta Imamura, Fernando Ezquerro, Fábio Marcon Alfieri, Lucy Vilas Boas, Tania Regina Tozetto-Mendoza, Janini Chen, Levent Özçakar, Lars Arendt-Nielsen, Linamara Rizzo Battistella

**Affiliations:** ^1^Clinical Research Center, Institute of Physical Medicine and Rehabilitation, University of São Paulo School of Medicine, Rua Jandiatuba, 580 Vila Andrade, 05716-150 São Paulo, SP, Brazil; ^2^University of São Paulo School of Medicine, Avenida Dr. Arnaldo, 455 Cerqueira César, 01246903 São Paulo, SP, Brazil; ^3^São Paulo Adventist University Center, Estrada de Itapecerica, 5859 Jardim IAE, 05858-001 São Paulo, SP, Brazil; ^4^Tropical Hematology Laboratory, Institute of Tropical Medicine of São Paulo and Medical Research Laboratory, LIM 52, University of São Paulo, Avenida Dr. Arnaldo, 455 Cerqueira César, 01246903 São Paulo, SP, Brazil; ^5^Institute of Physical Medicine and Rehabilitation, University of São Paulo School of Medicine, Rua Domingo de Soto 100, Vila Mariana, 04116-030 São Paulo, SP, Brazil; ^6^Department of Physical Medicine and Rehabilitation, Hacettepe University Medical School, Hacettepe University Faculty of Medicine, Sıhhiye, 06100 Ankara, Turkey; ^7^Center for Sensory-Motor Interaction, School of Medicine, Aalborg University, Fredrik Bajers Vej 7, Building D3, 9220 Aalborg E, Denmark

## Abstract

Osteoarthritis (OA) is the most common joint disorder in the world. Among the mechanisms involved in osteoarthritis, biomarkers (cytokines profile) may be related to pain and pain intensity, functional capacity, and pressure pain thresholds (PPT). Thus, the study of these relationships may offer useful information about pathophysiology and associated mechanisms involved in osteoarthritis. Therefore, the objective of this study was to investigate the seric concentration of pro (IL-6, IL-8, and TNF-*α*) and anti-inflammatory (IL-10) cytokines in patients with painful knee osteoarthritis and to correlate the levels of these biomarkers with the patients' functional capacity and pressure pain threshold (PPT) values.

## 1. Introduction

Osteoarthritis (OA) is the most common joint disorder in the world [1]. In the United States, the symptomatic knee OA occurs in 10% of the male and 13% of the female population with 60 years of age or more, with an estimated financial burden of US$ 47.8 billion annually [2, 3]. In Brazil, this disease is estimated to affect 6% to 12% of the adults with more than one third of those aged 65 years or over [4]. Due to the current trend of population aging and the increasing prevalence of obesity, it is estimated that the number of people suffering from knee OA will probably increase in the coming years [5] with substantial reduction in quality of life for the individual and massive costs for societies.

Different mechanisms may underlie the heterogeneous and multifactorial presentation of OA and inflammation is considered as one of the factors involved at some stages in the OA progression. The inflammatory process may be associated with alterations in the cytokines profile (e.g., interleukin-6) [6]. In addition, specific cytokines may provoke damage of the extracellular matrix of the joint tissue [7]. Some cytokines may act as biochemical markers of OA severity and pain [8]. Recent studies have indicated that specific pain mechanisms in OA may relate to biomarkers associated with cartilage and bone degradation [9]. Yet articular cartilage and subchondral bone damage are key factors in OA and can be assessed by a variety of new biomarkers [10] such as C-telopeptide of Type I collagen (CTX-I), CTX-II, Type III collagen N-propeptide, and matrix metalloproteinase (MMP) mediated degradation fragments of connective tissue turnover (C1M and C2M) and synovium (C3M) connective tissue turnover [11]. Ishijima et al. [12] showed that pain and Type II collagen degradation (sC2C and uCTX-II) and formation (sCPII), bone resorption (uNTx), and hence, for example, synovitis may contribute to joint pain. Another novel biomarker specific of MMP cleavage is the CRPM that reflects local inflammation, whereas circulating, uncleaved C-reactive protein (CRP) reflects systemic inflammation [13]. It is therefore of relevance to investigate how joint damage, measured by these novel disease related biomarkers, is related to pain mechanisms. Further, many authors have reported on the effects of different variables, such as functional performance [14], obesity [15], clinical aspects [16], and radiological severity of OA [6], on the serum or intra-articular levels of cytokines. In obesity, more and more studies have shown hypersensitivity to pain and that those individuals are more vulnerable to developing pain possibly due to the low inflammatory processes initiated by cytokine release from adipose tissue [17]. It has been already shown that pain in knee OA patients may be associated with generalized pain sensitization rather than peripheral inflammation and injury [18–21]. It is well known that joint damage in the individual OA patient is not strongly associated with pain [22, 23], suggesting many other factors are involved in driving the clinical manifestations in OA.

To further address this issue, the current study (1) investigated serum concentrations of proinflammatory cytokines (IL-6, IL-8, and TNF-*α*) and an anti-inflammatory cytokine (IL-10) in OA patients (with painful knee osteoarthritis) and healthy controls and (2) correlated these serum levels with pain intensity, functional capacity, and pressure pain thresholds (PPTs).

## 2. Method

This study was approved by the Ethics Committee for Analysis of Research Projects (CAPPesq), Hospital of Clinics of University of São Paulo Medical School, São Paulo, Brazil, research protocol CAPPesq 0131/10. All participants were informed about the study procedure and they all signed an informed consent. The procedures that followed were in accordance with the ethical standards of the responsible committee on human experimentation (institutional and national) and with the Helsinki Declaration of 1975, as revised in 2000.

### 2.1. Study Population

The recruitment of 101 subjects was done through referrals from various departments of the Hospital of Clinics and via contact with friends or family members who volunteered to participate.

Knee OA subjects (*N* = 53) were enrolled if they had the following: age ≥ 60 years, diagnosis of knee OA according to the American College of Rheumatology criteria and Kellgren/Lawrence scale grades of 2–4 [24, 25], and moderate to severe pain (visual analogue scale (VAS) > 4), as general averaged level of pain experienced during the day for the past month, lasting ≥ 3 months.

Healthy volunteers without any pain (*N* = 48) participated as controls. Those who had any rheumatic disease, fibromyalgia and other chronic painful condition, a history of malignancy, psychiatric disorder, or previous knee surgery were not included in the study.

### 2.2. Assessment of Function and Pain

Participants answered the Western Ontario and McMaster Universities Arthritis Index (WOMAC) questionnaire for the assessment of pain and function during daily activities [26].

Pain intensity was assessed using the 10 cm VAS ruler graded from zero (no pain) to ten (average pain intensity during the past 48 hours, during daily activities).

The pressure pain threshold (PTT) was measured by a pressure algometer (Pain Diagnostics, Great Neck, NY, USA) having a hard rubber 1 cm^2^ at the end of the apparatus. The pressure was applied perpendicular to the skin at a constant speed (1 Kg/s) and the patients indicated when they felt pain. The reading is expressed in kg/cm^2^.

Pressure pain thresholds were assessed over vastus medialis, adductor longus, rectus femoris, vastus lateralis, tibialis anterior, peroneus longus, iliacus, sartorius, gracilis, quadratus lumborum, and popliteus as previously described [18]. For this evaluation, the subject remained supine and prone and in the lateral position for better evaluation. We have not performed measures over the knee joints. In addition, PPT values were assessed over the ligaments located over the supraspinous ligaments (L) between L1-L2, L2-L3, L3-L4, L4-L5, and L5-S1 and sacral (S) areas S1-S2. During this assessment, the volunteer lied in the lateral decubitus position with hip flexion and the evaluator applied the pressure directly over the region. The pinch and roll maneuver at the L1, L2, L3, L4, L5, S1, and S2 dermatomes was done to evaluate subcutaneous hyperalgesia [27]. Except for the supraspinous ligaments and the L5–S1 and S1–S2 sacral areas (6 sites), all measurements were done bilaterally [28].

### 2.3. Blood Samples

Serum concentrations of proinflammatory cytokines (IL-6, IL-8, and TNF-*α*) and an anti-inflammatory cytokine (IL-10) were collected by blood samples (5 mL), taken from the antecubital vein between 8:00 am and 11:00 am. A Vacutainer tube, without the addition of anticoagulants, was used in order to obtain serum after the centrifugation process at 500 g, 4°C. Samples of sera were separated in 100 uL aliquots in Eppendorf cryotube and stored at −80°C until processing. Data was obtained by measuring the fluorescence intensity. Human Inflammatory Cytokines Kit (BDTM Bioscience CBA Cytometric Bead Array, San Jose, CA) was used to quantitatively measure serum concentrations of interleukin-6 (IL-6), interleukin-8 (IL-8), and tumor necrosis factor (TNF-*α*) according to the manufacturer's instructions. Individual cytokine concentrations (pg/mL) were computed using the standard reference curve of CELLQUEST and CBA software. The reading of the serum samples was performed by the cytometer FACS Calibur (BD Biosciences, USA) from the Laboratory of Medical Investigation (LIM 56), University of São Paulo School of Medicine. The serum measurements of proinflammatory cytokines (IL-6, IL-8, and TNF-*α*) and an anti-inflammatory cytokine (IL-10) were performed by an independent investigator blinded to the patients' status (knee osteoarthritis or healthy volunteers).

### 2.4. Statistics

Kolmogorov-Smirnov test and the observation of the morphology of the histogram were used to test for a normal distribution for all studied variables.

Quantitative characteristics with use of summary measures (median, interquartile interval) were compared between groups using Mann-Whitney *U* test. Correlations between age, pain duration, VAS, WOMAC scores, and the PPT values were evaluated using scatter plots and Spearman's rank correlation coefficients. Stepwise multiple linear regression models analyzed the relationships of VAS, WOMAC subscales, and the PPT measures. Napierian logarithm transformation was used to stabilize variability of serum cytokine values. Analyses were performed using SPSS software, version 15 (SPSS, Chicago, IL). *P* values less than 0.05 (2-tailed) were considered significant.

## 3. Results

Mean age values of symptomatic patients (71.23 ± 7.62 years, *n* = 53 (women)) and controls (68.21 ± 7.17 years, *n* = 48, 2 men and 46 women) were not significantly different (Table 1). All patients reported pain duration of at least 24 months (median, 96 months; range 36–150). About the use of drugs in the group of OA knee individuals, 36 were taking drugs, 22 with several medications such as antidepressants and antihypertensive, among other items, and 14 were using mediation analgesic. In the control group, 41 were taking several medications such as antidepressants and antihypertensive, among others.

### 3.1. Function and Pain

Mean WOMAC (pain, stiffness, and function) scores in the OA patients were 10.90 ± 2.35, 4.20 ± 2.35, and 42.53 ± 14.59, respectively. Mean pain intensity in the OA patients was 7.6 cm ± 1.59.

Knee OA patients showed lower PPTs compared to the matched controls (*P* < 0.05) in all 24 muscular, ligamentous, and subcutaneous points tested, both at the knees and at distant sites (Table 2).

### 3.2. Blood Samples

Serum levels of IL-8 and TNF-*α* were similar between patients and controls (Table 2). IL-6 (*P* = 0.031) and IL-10 (*P* = 0.030) levels were significantly elevated in patients (Table 3).

### 3.3. Correlation Analysis

Correlation analyses of variables are given in Table 4. Both IL-6 and IL-10 correlated positively with VAS and WOMAC scores for rigidity. Only IL-6 correlated positively with WOMAC scores for pain. IL-6 and IL-10 did not correlate with any PPT measurements.

PPTs from muscular, ligamentous, or subcutaneous sources showed the most consistent positive associations with TNF-*α* (Table 4).

For the pinch and roll cutaneous assessment, consistent positive associations were found with TNF-*α* (Table 4).

### 3.4. Regression Analysis

Stepwise analysis revealed that a combination of three variables added gain to the equation. Besides VAS, combined PPT values over pes anserinus and supraspinous ligaments at L1-L2 were the best predictors (*R* ^2^ = 0.244) for serum TNF-*α* (Tables 4 and 5). For serum IL-6, we found that WOMAC rigidity and VAS could be added to the equation (*R* ^2^ = 0.186) (Tables 4 and 5).

We could not find any predictors for serum IL-8 or IL-10 (Tables 4 and 5).

## 4. Discussion

### 4.1. Main Findings

The present cross sectional observational study (i) explored the serum concentration of proinflammatory cytokines (IL-6, IL-8, and TNF-*α*) and anti-inflammatory cytokines (IL-10) in patients with knee OA and healthy controls and (ii) associated those with clinical pain manifestations and pain hyperalgesia as assessed by PPTs and function. OA patients presented spreading sensitization as assessed by pressure pain hyperalgesia from all the structures investigated. IL-6 and IL-10 were higher in the knee OA patients compared with matched controls. As an exploratory study, we have not corrected for multiple analyses, and therefore, for 30 comparisons, two positive results can be by chance.

### 4.2. Cytokines Profile

Cytokines are glycoproteins of light molecular weight, responsible for the communication among cells of the immune system [29]. Several authors identified the correlation of higher levels of serum cytokines and knee OA, such as IL-6, TNF-*α*, and TNF-*α* soluble receptors sTNFR1 and sTNFR2, and C-reactive protein (CRP) [15, 30]. Classically, increased levels of proinflammatory cytokines are related to development and progression of OA due to upregulation of metalloproteinase gene expression, stimulation of reactive oxygen species production, alteration of chondrocyte metabolism, and increased osteoclastic bone reabsorption [15, 30–33].

Several authors have described the association of disability, measured by the WOMAC scores, with higher levels of proinflammatory cytokines [14, 15, 18, 34]. TNF has been compared to other proinflammatory cytokines and shown to be elevated in elderly patients with knee osteoarthritis [6, 35, 36]. Regarding knee OA, the most commonly measured cytokines in the literature are IL-6 and TNF-*α* [6, 37, 38]. Similar to previous reports [6, 35, 39], we observed that serum levels of IL-6 had a twofold increase in patients when compared to controls, comparable to a study that showed increased IL-6 in patients with more severe disease as measured by radiologic knee OA findings [6]. On the other hand, serum concentrations of the other cytokines are very heterogeneous to draw any conclusions. We also demonstrated that symptomatic patients presented less resistance to algometer-induced PPT than did the healthy controls in all 24 muscular, ligamentous, and subcutaneous points tested, not only at the knees. Both IL-6 and IL-10 correlated positively with VAS and WOMAC scores for rigidity. Only IL-6 correlated positively with WOMAC pain scores.

In fact, many have investigated the effect of different presentations of OA on the serum or intra-articular levels of cytokines [6, 14–16]. However, to the best of our knowledge, this is the first report regarding the association between serum proinflammatory cytokines and PPTs that are located further beyond the painful area at the involved joint. This indicates a sclerotome-wide phenomenon that can be explained only neurologically. On the other hand, the lack of relation between this particular cytokine and pain duration, WOMAC scores, or VAS remains to be clarified. Additionally, since cytokine IL-10 was not detectable in the cerebrospinal fluid of patients with knee OA, the concept of central sensitization as an important mechanism in the causal chain of disability would be noteworthy [40]. Several authors have already demonstrated that proinflammatory mediators as interleukins are increased in fibromyalgia patients [41, 42]. It seems that chronic pain, hyperalgesia, and fatigue are associated with increased levels of IL-6 and IL-8 in these patients [18, 41]. Similarly, tissue lesion may, in most cases, not be the major issue determining loss of function and quality of life in OA.

Interestingly, muscle tender points and lower tolerance to pressure areas have higher levels of TNF-*α* when compared to healthy individuals without spontaneous pain or asymptomatic subjects [43–45]. In subjects with spontaneous pain, areas distant from the site of pain also showed a significant increase of these chemical mediators [45]. Cytokine production has also been demonstrated in nonimmune tissue under special conditions such as acute inflammation. Production of proinflammatory cytokines such as IL-1-*β*, IL-6, and TNF-*α* has recently been found in skeletal muscle tissue during special conditions such as endotoxemia [46]. However, the influence of hyperalgesia found in muscle tissues of patients with OA on serum cytokines has not been determined, although leaking from local microenvironment could be a possible mechanism.

Still, we must remember that the process called inflammaging indicating a regulation of the inflammatory response that occurs with aging can result in the production of inflammatory cytokines, which generates a low level of chronic and systemic proinflammatory state [47, 48]. This may occur due to the presence of chronic diseases associated with aging such as OA, as well as other conditions such as obesity and physical inactivity [47].

A possible source of local cytokine production, different from the inflammatory cells, may be the adipose tissue. It is known that adipose tissue produces and secretes several proinflammatory cytokines, including IL-6 and TNF-*α* [49]. Despite the lack of correlations between IL-6 and the PPT values over any of the assessed tissues, we observed that PPT value over supraspinous ligament L1-L2 combined with pes anserinus and VAS can predict serum levels of TNF-*α*. Yet increased production and secretion of proinflammatory cytokines (biomarkers) by adipose cells have been hypothesized as the role played by obesity as a risk factor for OA [50]. According to Livshits et al. [6], when inflammation occurs, cytokine profile changes and seems to be influenced by obesity and aging [15, 16]. In fact, we have recently demonstrated that aging may play a role in cytokine profile, a finding not so extensively addressed in the literature and that should be further investigated [51]. In the elderly population, body metabolism and composition, as well as the response to acute inflammation, may be altered and influenced in a different pattern by cytokines than in the acute-phase response, all of which are altered with increasing age [52]. TNF-*α* seems to act as a biomarker of physiologic status and a major predictor of strength and functional status in the elderly [6]. IL-1*α*, IL-1*β*, and TNF-*α* seem to induce production of IL-6 [52]. To the best of our knowledge, the contribution of inflammatory factors such as IL-6 and TNF-*α* to the involvement of structures outside the knee joint has not been adequately explored yet, in particular, the correlation of structures that are innervated by the same spinal nerve constituting the metameres.

Our study had some limitations. First, we did not measure other cytokines such as IL-1 or IL-Ra, due to the budget of the study. Another item is the lack of radiographic evaluation of the control subjects. But it is noteworthy that, in the clinical evaluation of these patients, absence of pain was observed and evaluated. However, further studies with radiographic classification of healthy volunteers, with other cytokines, are needed to confirm our findings and to explore the local production of cytokines by skeletal muscle (myocytes), adipose cells, and the surrounding connective tissue.

## 5. Conclusion

This study showed that serum levels of IL-6 and IL-10 were higher in knee OA than in healthy controls. A positive association between IL-10 and IL-6 with VAS and WOMAC for pain and rigidity scores was found. These findings align with a physiopathological understanding of pain and functionality that surpasses the one which currently provides basis for therapeutics in OA.

## Figures and Tables

**Table 1:** Comparison of age and BMI (body mass index) between the knee osteoarthritis (OA) and healthy control groups.

	Knee OA	Control	*P*
*N*	53	48	
Age (years)	71.23 ± 7.62	68.21 ± 7.17	0.066
BMI (kg/cm^2^)	28.33 ± 5.95	27.42 ± 4.86	0.55

Note: values are presented as mean and standard deviation (SD). Kg: kilogram. Cm: centimeter. BMI: body mass index.

**Table 2:** Pressure pain threshold (PPT) values from the assessed structures.

	Group										
Variable	Healthy control			Knee OA			Mean difference	CI (95%)		*P*	Cohen's *d*
	Mean	SD	*N*	Mean	SD	*N*		Lower	Upper		
Vastus M	6.00	2.08	48	3.30	1.81	53	2.70	1.93	3.47	**<0.001**	1.38
Adductor L	4.97	2.20	48	2.73	1.44	53	2.24	1.51	2.97	**<0.001**	1.21
Rectus fem	7.44	2.29	48	4.19	2.18	53	3.25	2.37	4.13	**<0.001**	1.45
Vastus L	6.13	2.09	48	3.47	2.10	53	2.66	1.83	3.49	**<0.001**	1.27
Tibialis ant	7.43	2.53	48	4.04	2.10	53	3.39	2.48	4.31	**<0.001**	1.46
Peroneus	7.40	2.33	48	4.21	2.96	53	3.19	2.13	4.25	**<0.001**	1.20
Gracilis	5.97	2.47	48	2.82	1.89	53	3.15	2.28	4.01	**<0.001**	1.43
Iliopsoas	6.45	3.36	48	3.50	1.34	53	2.95	1.96	3.95	**<0.001**	1.15
Quadratus L	6.86	2.04	48	4.10	1.86	53	2.77	2.00	3.54	**<0.001**	1.42
Pes anserinus	5.40	2.49	48	2.75	2.01	53	2.65	1.76	3.55	**<0.001**	1.17
Popliteus	7.05	2.36	48	3.87	2.20	53	3.18	2.28	4.08	**<0.001**	1.39
Lig L1-L2	7.98	3.12	48	4.46	2.07	53	3.52	2.48	4.55	**<0.001**	1.33
Lig L2-L3	8.38	3.11	48	4.37	2.21	53	4.01	2.95	5.06	**<0.001**	1.49
Lig L3-L4	8.09	3.10	48	4.58	2.31	53	3.51	2.44	4.58	**<0.001**	1.29
Lig L4-L5	8.29	2.89	48	4.64	2.10	53	3.65	2.66	4.64	**<0.001**	1.44
Lig L5-S1	8.70	2.98	48	4.42	2.27	53	4.28	3.24	5.32	**<0.001**	1.62
Lig S1-S2	8.72	3.21	48	4.90	2.81	53	3.82	2.63	5.01	**<0.001**	1.27
PRL1	3.79	2.11	48	2.39	3.45	53	1.40	0.25	2.54	**<0.001**	0.40
PRL2	3.67	1.83	48	1.77	1.33	53	1.90	1.27	2.53	**<0.001**	1.19
PRL3	4.26	1.74	48	2.01	1.34	53	2.25	1.64	2.86	**<0.001**	1.45
PRL4	5.58	2.17	48	2.95	1.77	53	2.63	1.85	3.41	**<0.001**	1.33
PRL5	5.09	1.98	48	2.47	1.58	53	2.62	1.92	3.33	**<0.001**	1.46
PR S1	5.51	1.59	48	3.02	1.59	53	2.50	1.87	3.12	**<0.001**	1.57
PR S2	4.80	1.92	48	2.77	1.49	53	2.03	1.35	2.70	**<0.001**	1.18

OA: osteoarthritis; Vastus M: vastus medialis; Adductor L: adductor longus; Rectus fem: rectus femoris; Vastus L: vastus lateralis; Tibialis ant: tibialis anterior.

PR: pinch and roll maneuver; Lig: supraspinous ligaments; Quad L: quadratus lumborum; L: lumbar; S: sacral.

SD: standard deviation; CI: confidence interval; *N*: number; *P*: probability of significance.

**Table 3:** Comparison of cytokine concentrations between groups.

Variable	Group	Mean	SD	Median	Quartile 1	Quartile 3	*N*	*P*
TNF*α* (pg/mL)	Healthy	1.40	1.02	1.29	0.0	2.8	48	0.457
	Knee OA	2.20	4.52	1.30	1.1	1.5	53	
	All	1.82	3.36	1.26	0.0	24.2	101	

IL-10 (pg/mL)	Healthy	0.89	0.87	1.30	0.0	2.3	48	**0.030**
	Knee OA	1.58	2.36	1.17	0.0	1.2	53	
	All	1.25	1.84	1.09	0.0	15.7	101	

IL-6 (pg/mL)	Healthy	2.55	1.06	2.9	2.3	4.0	48	**0.031**
	Knee OA	4.38	4.61	2.4	1.7	3.1	53	
	All	3.48	3.53	2.50	1.2	19.3	101	

IL-8 (pg/mL)	Healthy	9.72	5.28	8.80	5.4	16.0	48	0.984
	Knee OA	10.56	8.51	9.00	6.2	12.7	53	
	All	10.16	7.14	8.83	0.0	53.5	101	

OA: osteoarthritis; Healthy: healthy control; IL: interleukin; TNF: tumor necrosis factor; SD: standard deviation; *N*: number of subjects; *P*: probability.

**Table 4:** Correlation among cytokines, age, pain duration, VAS, WOMAC scores, and PPT values over 24 anatomical structures.

Variables		TNF-*α*	IL-10	IL-6	IL-8
Age	Spearman^*^	−0.154	−0.019	0.016	−0.006
	*P*	0.123	0.854	0.873	0.954
	*N*	101	101	101	101

Pain duration (months)	Spearman^*^	−0.039	0.185	0.166	−0.001
	*P*	0.706	0.070	0.105	0.993
	*N*	97	97	97	97

VAS	Spearman^*^	−0.029	0.203	0.238	0.020
	*P*	0.774	0.042	0.017	0.842
	*N*	101	101	101	101

WOMAC pain	Spearman^*^	−0.145	0.177	0.251	0.019
	*P*	0.151	0.078	0.012	0.850
	*N*	100	100	100	100

WOMAC rigidity	Spearman^*^	−0.050	0.271	0.303	0.059
	*P*	0.626	0.007	0.002	0.562
	*N*	99	99	99	99

WOMAC difficulties	Spearman^*^	−0.134	0.166	0.192	0.013
	*P*	0.182	0.097	0.055	0.896
	*N*	101	101	101	101

Vastus M	Spearman^*^	0.283	0.075	−0.070	0.113
	*P*	0.004	0.453	0.488	0.259
	*N*	101	101	101	101

Adductor L	Spearman^*^	0.337	0.054	−0.076	0.097
	*P*	0.001	0.589	0.449	0.335
	*N*	101	101	101	101

Rectus A	Spearman^*^	0.229	0.034	−0.056	0.133
	*P*	0.021	0.737	0.580	0.185
	*N*	101	101	101	101

Vastus L	Spearman^*^	0.252	−0.006	0.015	0.154
	*P*	0.011	0.955	0.880	0.124
	*N*	101	101	101	101

Tibialis ant	Spearman^*^	0.236	−0.067	−0.065	0.096
	*P*	0.017	0.507	0.519	0.338
	*N*	101	101	101	101

Peroneal	Spearman^*^	0.322	0.001	−0.075	0.067
	*P*	0.001	0.992	0.456	0.503
	*N*	101	101	101	101

Pes anserinus	Spearman^*^	0.339	0.113	0.009	0.099
	*P*	0.001	0.259	0.932	0.326
	*N*	101	101	101	101

Gracilis	Spearman^*^	0.322	0.050	−0.031	0.101
	*P*	0.001	0.623	0.761	0.316
	*N*	101	101	101	101

Iliopsoas	Spearman^*^	0.136	−0.098	−0.060	−0.058
	*P*	0.175	0.330	0.554	0.564
	*N*	101	101	101	101

Quad L	Spearman^*^	0.228	−0.080	−0.087	−0.030
	*P*	0.022	0.424	0.384	0.764
	*N*	101	101	101	101

Lig L1-L2	Spearman^*^	0.204	−0.122	−0.032	0.084
	*P*	0.041	0.223	0.750	0.406
	*N*	101	101	101	101

Lig L2-L3	Spearman^*^	0.192	−0.100	−0.031	0.034
	*P*	0.055	0.322	0.762	0.733
	*N*	101	101	101	101

Lig L3-L4	Spearman^*^	0.139	−0.117	−0.068	0.037
	*P*	0.165	0.243	0.496	0.711
	*N*	101	101	101	101

Lig L4-L5	Spearman^*^	0.187	−0.091	−0.034	0.098
	*P*	0.061	0.367	0.737	0.330
	*N*	101	101	101	101

L5-S1	Spearman^*^	0.188	−0.045	−0.048	0.102
	*P*	0.060	0.656	0.635	0.312
	*N*	101	101	101	101

Lig S1-S2	Spearman^*^	0.171	−0.100	−0.067	0.081
	*P*	0.087	0.318	0.504	0.420
	*N*	101	101	101	101

Popliteus	Spearman^*^	0.266	−0.102	−0.067	0.056
	*P*	0.007	0.308	0.503	0.581
	*N*	101	101	101	101

PRL1	Spearman^*^	0.328	0.089	−0.077	0.057
	*P*	0.001	0.377	0.445	0.574
	*N*	101	101	101	101

PRL2	Spearman^*^	0.323	0.052	−0.063	0.123
	*P*	0.001	0.607	0.529	0.221
	*N*	101	101	101	101

PRL3	Spearman^*^	0.313	0.017	−0.046	0.059
	*P*	0.001	0.870	0.645	0.559
	*N*	101	101	101	101

PRL4	Spearman^*^	0.253	−0.061	−0.094	0.138
	*P*	0.011	0.547	0.349	0.169
	*N*	101	101	101	101

PRL5	Spearman^*^	0.278	−0.049	−0.013	0.073
	*P*	0.005	0.627	0.896	0.470
	*N*	101	101	101	101

PR S1	Spearman^*^	0.275	−0.104	−0.151	−0.026
	*P*	0.005	0.303	0.132	0.798
	*N*	101	101	101	101

PR S2	Spearman^*^	0.221	0.000	−0.068	−0.008
	*P*	0.026	0.998	0.497	0.938
	*N*	101	101	101	101

^*^Spearman's rank correlation coefficients.

Vastus M: vastus medialis; Adductor L: adductor longus; Rectus A: rectus femoris; Vastus L: vastus lateralis; Tibialis ant: tibialis anterior; PR: pinch and roll maneuver; Lig: supraspinous ligaments; Quad L: quadratus lumborum; L: lumbar; S: sacral; IL: interleukin; TNF: tumor necrosis factor; WOMAC: Western Ontario and McMaster Universities Arthritis Index; *r*: Spearman correlation; *P*: probability.

**Table 5:** Stepwise multiple linear regression models for IL-6 and TNF-*α* and age, VAS, WOMAC pain subscale, WOMAC rigidity subscale, WOMAC physical activity subscale, and pressure pain threshold values (in kg/cm^2^) in 24 studies structures.

IL-6 (pg/mL)	*R* ^2^	0.186	*P*
Independent variables	Estimated parameter	Standard error	
(Constant)	0.961	0.125	<0.000
WOMAC rigidity	0.157	0.034	<0.000
VAS L	0.050	0.019	0.012

TNF-*α* (pg/mL)	*R* ^2^	0.244	*P*
Independent variables	Estimated parameter	Standard error	

(Constant)	0.356	0.189	0.062
Pes anserinus	0.167	0.033	0.000
VAS	0.049	0.018	0.007
Ligaments L1-L2	−0.059	0.027	0.032

L: left.
